# Surgical retrieval of a swallowed denture in a schizophrenic patient: a case report

**DOI:** 10.1186/s13037-017-0147-8

**Published:** 2017-12-18

**Authors:** Johannes Dörner, Herbert Spelter, Hubert Zirngibl, Peter C. Ambe

**Affiliations:** Department of Surgery, HELIOS Universitätsklinikum Wuppertal, Witten – Herdecke University, Heusnerstr. 40, 42283 Wuppertal, Germany

## Abstract

**Background:**

Accidental foreign body ingestion is a common phenomenon in children between 6 months to 6 years of age. In adults, foreign body ingestion is commonly observed in the geriatric population and in patients with psychiatric disorders. Over 80% of ingested foreign bodies pass uneventfully through the intestinal tract. Endoscopic retrieval is needed in about 20% while surgical intervention is indicated in less than 1%. Herein we report an extremely rare case of esophagocutaneous fistula following operative retrival of an impacted denture in the esophagus with spontaneous healing within 3 weeks. A similar case to the best of our knowledge has so far not been reported previously.

**Case presentation:**

A case of accidental ingestion of a dental prosthesis in a 35-year old schizophrenic patient is presented. The patient was referred to our department after accidentally swallowing one of his dental prosthesis. Surgical retrieval was indicated after two unsuccessful endoscopic retrieval attempts**.** The denture was retrieved following a longitudinal incision of the esophagus via a left cervical approach. The postoperative course was complicated by a clinically suspected esophagocutaneous fistula which was managed conservatively via nothing per os with enteral feeding via a nasogastric tube. Secretion ceased 3 weeks later and a fistula could not be found on contrast enhanced radiographic examination with gastrographin®.

**Conclusion:**

Esophagocutaneous fistula represents a rare but serious complication following foreign body ingestion. An interdisciplinary management including an early surgical consultation should be considered in patients with foreign body impaction in the esophagus following failure of endoscopic retrieval.

## Background

Accidental foreign body (FB) ingestion is a common phenomenon in children between 6 months to 6 years of age. In adults, foreign body ingestion is commonly observed in the geriatric population and in patients with psychiatric disorders. In the adult population, fishbone, small animal bones, food bolus and dental work represent the most commonly ingested FB [1]. FB-associated complications might be secondary to impaction. Physiologically narrow areas along the gastrointestinal tracts predispose to FB impaction [2]. The esophagus is involved in about 75% of cases [3, 4]. FB ingestion might cause symptoms like choking, anorexia, hypersalivation, wheezing or respiratory distress. Diagnostic workup usually begins with a careful history and physical examination aimed at localizing the FB and excluding serious complications such as impaired airway, peritonitis and small bowel obstruction. Imaging studies, most commonly a radiography of the neck, thorax and or abdomen may be performed, not only to localize the FB but also to gain information about its physical properties (size, shape, etc). Besides, the position of the FB and eventually complications such as perforation leading to pneumomediastinum or pneumoperitoneum might be evident on radiographic images. However, some ingested FBs are not radiopaque, so that endoscopic examination via esophagogastroduodenoscopy (EGD) might be indicated. Although the majority of FBs pass the gastrointestinal tract uneventfully, endoscopic retrieval is required 20% of cases [5]. Surgery is indicated in about 1% following failure of endoscopic retrieval or to address complications [4]. Herein we present an extremely rare case of postoperative esophagocutaneous fistula following operative retrieval of an impacted denture in the esophagus with spontaneous fistula closure within 3 weeks.

## Case presentation

**Fig. 1:**
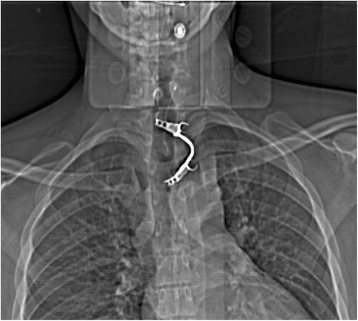
Radiograph showing the ingested denture in the proximal esophagus

**Fig. 2:**
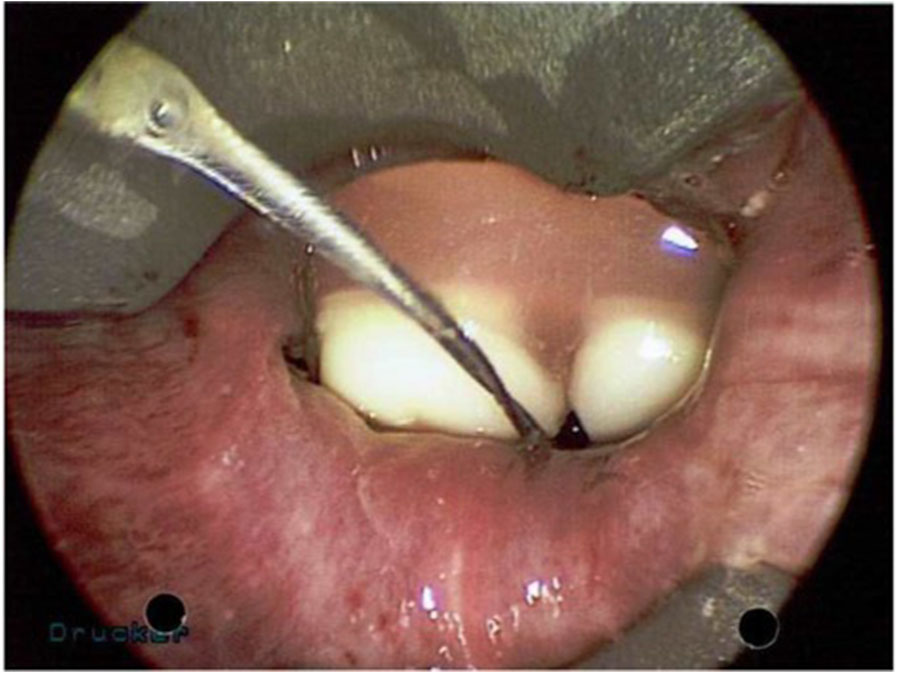
Endoscopic image during rigid laryngoscopy

**Fig. 3:**
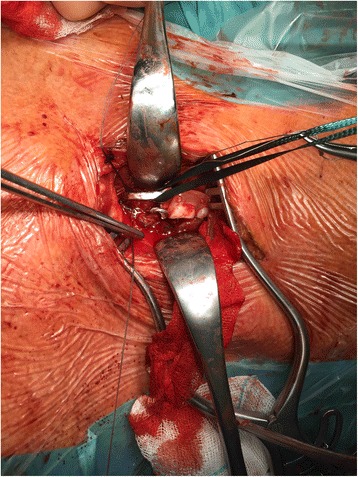
Intraoperative finding

**Fig. 4:**
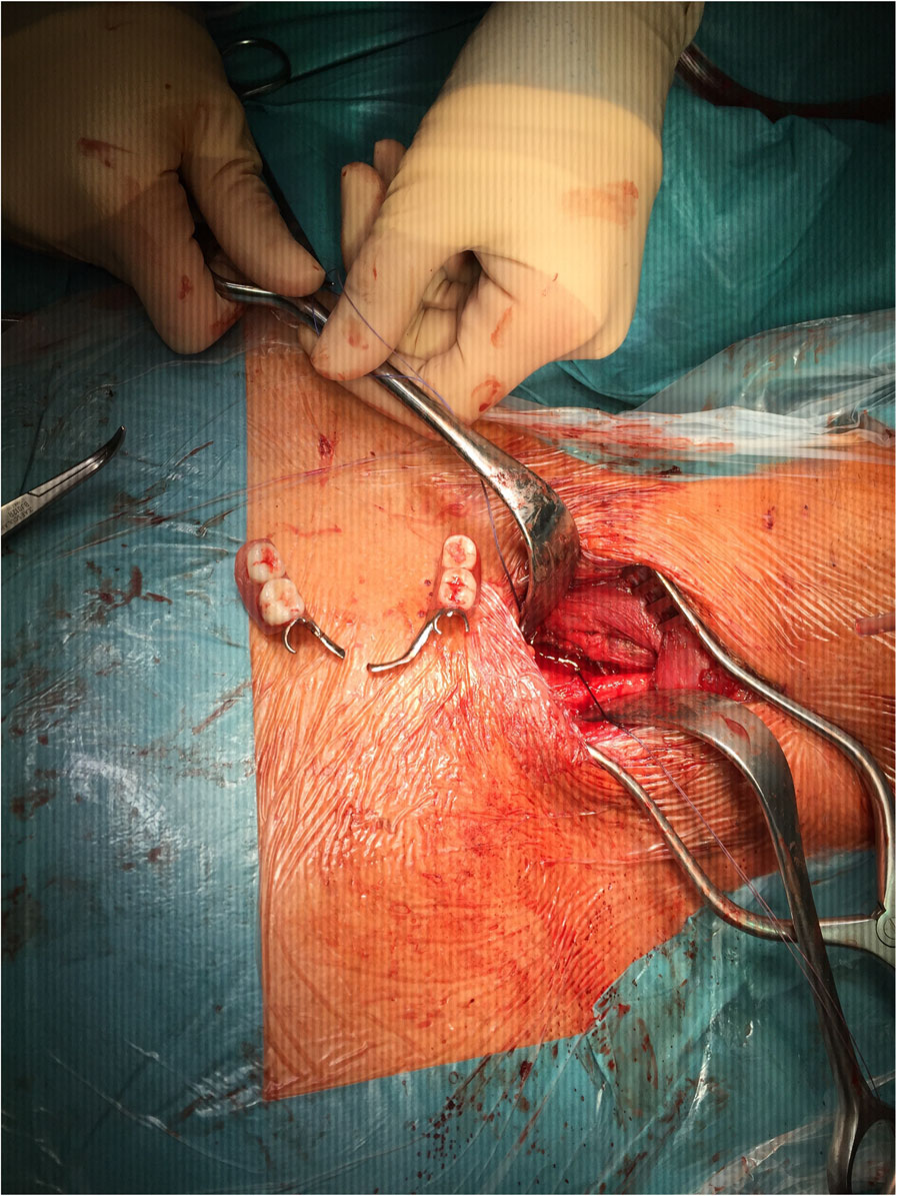
Extracted denture

A 35-year old caucasian schizophrenic patient was referred to our tertiary care center from another hospital after swallowing one of his dentures. Two endoscopic retrieval attempts were unsuccessful prior to referral. The patient complained of hoarseness and tenderness around the neck. A FB could be palpated at the left lateral side of the trachea just cranial to the jungulum. The denture was identified at the level of the cervical esophagus on x-ray **(**Fig. 1). No signs of perforation were seen on computed tomography (Fig. 2). Rigid laryngoscopy revealed an impaction of the dental prosthesis in the esophageal mucosa. The oesophagus was opened via a left cervical approach. The denture was found impacted in the esophageal wall following mucosal perforation via the protruding metallic holding clamps (Figs. 3 and 4). Thus, the denture was transected to enable retrieval without additional trauma. The esophageal wall was closed with a running suture and a drain was placed. Surgery was uneventful. However, the postoperative course was complicated by a clinically suspected esophagocutaneous fistula due to high volume output via the drain. However, no major leakage was seen on contrast enhanced radiographic examination (with gastrographin®). The patient was managed with nothing per os (NPO and feeding via a gastric tube until secretion ceased. Full recovery was achieved 3 weeks after surgery.

## Discussion

Foreign body ingestion is a relatively common emergency and the vast majority of cases can be managed conservatively or endoscopically. Surgical intervention is required in less than 1% of cases. Failure to retrieve a FB during endoscopy, FB impaction with bowel obstruction and bowel perforation represent the most common indications for surgery [6].

The case of a 35-year old schizophrenic patient who accidentally ingested one of his dentures is reported. The patient was referred to your tertiary care unit following two unsuccessful endoscopic retrieval attempts. The denture was found impacted in the cervical esophagus without radiologic signs of perforation. The case was discussed in an interdisciplinary setup with endoscopists and ENT specialists. A rigid posterior laryngoscopy was undertaken. The denture was found to be impacted in the esophageal wall. Surgery was indicated following failure of endoscopic retrieval.

The esophagus was exposed via a left cervical incision. The FB was reached and removed following a longitudinal esophagotomy. Surgery was uneventful. However, the postoperative course was complicated by an esophagocutenous fistula, which was conservatively managed.

The most relevant issue about this case is the physical nature of the FB. Generally, FB can be characterized with regard to their physical features including the nature of the edges. The pathomechanism in this case is associated with the physical features of the denture. The sharp edges of the denture holdings got impacted in the esophageal mucosa rendering endoscopic retrieval impossible. Besides, the time interval between FB ingestion and retrieval attempt might have played a role in this case [7]. Prolonged impaction might have led to pressure ulcers in the impacted region. This might predispose to perforation with mediastinitis and fistula formation. In fact, the postoperative course in this case was complicated by a suspected clinical non-relevant esophagocutaneous fistula. This complication was suspected following increased and prolonged secretion via the drain. However, a relevant leak could not be identified following contrast enhanced radiographic examinations. The spontaneous ceasation of secretion 3 weeks after surgery was interpreted as the result of a spontaneous closure of a non - significant fistula. Radiographs with contrast media failed to show any fistula. Such a complication might have been a sequela of esophageal ulceration following prolonged impaction.

Impacted FBs are generally difficult to retrieve. The use of an overtube or by fitting the endoscope with a protector hood has been propagated in such cases [8]. However, an impacted FB cannot always be removed without causing further damage. It is conceivable that a sharp FB further impacts in the hours following ingestion. Therefore sharp objects in the esophagus should be managed as a medical emergency and treatment should be initiated promptly [9]. Finally, dentures that carry sharp edges should be avoided in populations at risk for FB ingestion if possible.

## Conclusion

Esophagocutaneous fistula represents a rare but serious complication following foreign body ingestion. An interdisciplinary management including an early surgical consultation should be considered in patients with foreign body impaction in the esophagus following failure of endoscopic retrieval.

## Abbreviations

EGD: EsophagoGastroDuodenoscopy
FB: Foreign Body

## References

[1] Ambe P, Weber SA, Schauer M, Knoefel WT (2012). Swallowed foreign bodies in adults. Dtsch Arztebl Int.

[2] Sung SH, Jeon SW, Son HS, Kim SK, Jung MK, Cho CM, Tak WY, Kweon YO (2011). Factors predictive of risk for complications in patients with oesophageal foreign bodies. Dig Liver Dis.

[3] Mosca S, Manes G, Martino R, Amitrano L, Bottino V, Bove A, Camera A, De Nucci C, Di Costanzo G, Guardascione M (2001). Endoscopic management of foreign bodies in the upper gastrointestinal tract: report on a series of 414 adult patients. Endoscopy.

[4] Webb WA (1995). Management of foreign bodies of the upper gastrointestinal tract: update. Gastrointest Endosc.

[5] Ginsberg GG (1995). Management of ingested foreign objects and food bolus impactions. Gastrointest Endosc.

[6] Hong KH, Kim YJ, Kim JH, Chun SW, Kim HM, Cho JH (2015). Risk factors for complications associated with upper gastrointestinal foreign bodies. World J Gastroenterol: WJG.

[7] Peng A, Li Y, Xiao Z, Wu W (2012). Study of clinical treatment of esophageal foreign body-induced esophageal perforation with lethal complications. Eur Arch Otorhinolaryngol.

[8] Bertoni G, Sassatelli R, Conigliaro R, Bedogni G (1996). A simple latex protector hood for safe endoscopic removal of sharp-pointed gastroesophageal foreign bodies. Gastrointest Endosc.

[9] Ikenberry SO, Jue TL, Anderson MA, Appalaneni V, Banerjee S, Ben-Menachem T, Decker GA, Fanelli RD, Fisher LR, Fukami N (2011). Management of ingested foreign bodies and food impactions. Gastrointest Endosc.

